# Engineering circular RNA regulators to specifically promote circular RNA production

**DOI:** 10.7150/thno.56990

**Published:** 2021-05-24

**Authors:** Yangfan Qi, Wei Han, Dan Chen, Jinyao Zhao, Lu Bai, Fang Huang, Zhenwei Dai, Gang Li, Chaoqun Chen, Wenjing Zhang, Jinrui Zhang, Bilian Jin, Yang Wang

**Affiliations:** 1 Second Affiliated Hospital, Institute of Cancer Stem Cell, Dalian Medical University, Dalian, China 116044.; 2 Department of Pathology, First Affiliated Hospital, Dalian Medical University, Dalian, China 116044.

**Keywords:** circRNA, back-splicing, PUF domain, dimerization, ECRRs

## Abstract

**Background:** A large number of circular RNAs (circRNAs) have been discovered in the mammalian transcriptome with high abundance, which play vital roles in gene regulation, thereby participating in the development of multiple diseases. However, the biogenesis, regulation, and especially manipulation of circRNAs still remain largely unknown.

**Methods:** Engineering circRNA regulators (ECRRs) were developed to promote circRNA biogenesis. Multiple circRNA mini-gene reporters were generated to evaluate the regulatory role of ECRRs. RT-PCR, qRT-PCR, northern blot, western blot, and flow cytometry assays were applied to assess the efficiency of artificial circRNA regulators on circRNA production in the presence or absence of RNase R treatment.

**Results:** We engineered circRNA regulators by combining sequence-specific RNA binding motifs of human Pumilio 1 with functional domains that could form dimerization. We applied these engineered regulators to promote the circRNA production of the exogenous circRNA minigene reporter circGFP, thereby stimulating the functional GFP protein generation. Crucially, such regulation is in time-course dependent and dose-dependent manners with designed specificity. Moreover, the application of ECRRs could also stimulate circRNA biogenesis of another minigene reporter circScreen, suggesting that ECRRs can be commonly used to promote circRNA generation of exogenous reporters. Most importantly, ECRRs could be utilized to specifically promote the production of the endogenous circRNAs circ10720 and circBIRC6 as well.

**Conclusion:** Our approach allows the creation of engineered regulators to target virtually any pre-mRNA *in vivo*, offering a novel avenue to investigate circRNA biogenesis and manipulate disease-related circRNA production.

## Introduction

Circular RNAs (circRNAs), a class of evolutionarily conserved non-coding RNAs, have been discovered in higher eukaryotes for more than two decades 1, 2. CircRNAs used to be regarded as byproducts of aberrant splicing only with a few functions 2, 3. With the development of the next-generation sequencing technique, a great number of circRNAs have been revealed in the mammalian transcriptome with high abundance 4-7, suggesting that many circRNAs might be functional. However, the functions of circRNAs still remain largely unknown. Several studies demonstrated that circRNAs could serve as microRNA sponges to control gene expression 5, 8. CircRNAs are capable of promoting Pol II transcription of their parental genes with unknown mechanisms 9. In addition, recent studies have discovered that N^6^-methyladenosine (m^6^A), the most abundant base modification of RNA, could promote efficient initiation of protein translation from circRNAs in human cells 10. Most importantly, circRNAs are able to control the progression of various human diseases, including atherosclerosis, nervous system disorders, diabetes, and cancer 11-15.

CircRNAs are mainly generated from pre-mRNA back-splicing, which is also processed by the spliceosome and regulated by *cis*-regulatory elements and *trans*-acting factors 9, 16, 17. Multiple studies demonstrated that circularization signals are detected in the introns flanking the circularized exons 4, 18, 19. The abundance of most circRNAs is quite lower, which is because the sterically unfavorable ligation of a downstream 5′ ss with an upstream 3′ ss by the spliceosome makes the lower efficiency of back-splicing than that of canonical splicing. Nevertheless, the abundant circRNAs always contain introns that are enriched in reverse complementary matches (such as ALU repeats), which could bring the distal splice site into close proximity to facilitate circRNA biogenesis 4, 18. In addition, RNA Binding Proteins (RBPs) could also promote circRNA production in distinct systems and organisms, including MBNL1, ADAR, DHX9, NF90/NF110, and so on 20-23. Thus, manipulation of the circRNA biogenesis will improve our understanding of circRNA regulation and might provide therapeutic potential for circRNA-involved diseases.

The approaches that could promote the production of circRNAs are still limited. Currently, a variety of vectors containing circRNA-producing exons and their flanking introns with intronic complementary sequences have been applied to introduce circRNAs to cells by transfection, which is similar to the overexpression of linear RNAs 18, 21, 23. However, such overexpression approach is usually accompanied by a great number of pre- and mature linear RNA isoforms. In addition, replacement of the weak promoter with a strong one using genome-editing tools could promote RNA products, nevertheless, such manipulation is able to elevate the production of both linear and circular RNAs. Moreover, RNA binding proteins, such as MBL and Quaking (QKI), could also promote circRNA production by binding to the flanking introns and stimulating intron-intron interactions, but the binding sites of MBL and QKI are needed 19,24. Therefore, flexible artificial modulatory tools of the circRNA biogenesis are urgently required.

Here we report the first attempt to develop a set of circRNA regulators with designed specificities. Such engineering circRNA regulators (ECRRs) are constructed by combining sequence-specific RNA-binding domains of human Pumilio1 (PUF domain) with functional domains that can form a homodimer. Importantly, we applied the ECRRs to specifically promote the back-splicing of both circRNA minigene reporters and the endogenous circRNAs. Our study provides a novel and effective approach to modulate the production of circRNAs*,* which has enormous application value in investigating the functions of different circRNAs, thereby offering great therapeutic potential for circRNA-related diseases.

## Materials and Methods

### Plasmids

To construct ECRRs, we fused the PUF domain of human Pumilio1 with different domains that could form homodimers. Complementary DNA encoding human QKI, CASTOR1, hnRNP A1, PRKAK1A and ZBTB18 were generated by PCR amplification and sub-cloned into pCI-neo vector using *Mlu* I/*Not* I restriction sites. The corresponding PUF domain with an N-terminal Flag epitope and a fragment encoding a nuclear localization sequence (NLS: PPKKKRKV) was amplified by primers described in Table S1, and subsequently ligated into the pCI-neo vector by *Xho* I/*Mlu* I sites. The resulting construct expresses an NLS-FLAG-PUF-type ECRR under the control of a CMV (Cytomegalovirus) promoter. To test the function of dimerization domain, we obtained the RRM domain of hnRNP A1, the BTB domain of ZBTB18, the GTPase domain of ATL1, the DH domain of ITSN, PRKAK1A(1-136 aa), and PKD1(47-178 aa), then respectively replaced hnRNPA1 of ECRR expression vector using *Mlu* I/*Not* I sites. The HA-tag ECRRs were amplified by HA-NLS foward primer that includes HA nucleotide sequence and NLS (nuclear localization signal) sequence (ccgCTCGAGccatgTACCCATACGACGTCCCAGACTACGCTCCCAAGAAAAAGAGGAAG) with reverse primers of different dimerization protein or domains from flag-tag ECRRs and subcloned into pCI-neo vector by *Xho* I/*Not* I sites. To promote the circRNA production of circScreen reporter, we cloned the domain PUF-L or PUF-R that only recognized the upstream or downstream intron target sequence in the reporter, then ligated into pCI-GTPase or pCI-BTB domain with *Xho* I/*Mlu* I sites. The PUF L-R were PUF-L and PUF-R plasmids co-transfected into the cells. To modulate the biogenesis of endogenous circRNA, PUF_10720_-GTPase, PUF_10720_-BTB, PUF_BIRC6_-GTPase or PUF_BIRC6_-BTB was cloned into pLVX-Puro vector with *Sma* I/*Not* I restriction sites. The point mutations were introduced into PUF domains for constructing different ECRRs through multistep PCR with site-directed primers. The sequence GGGCUGCA shared in flanking introns on both sides of the GFP exons was chosen as the ECRR target of circGFP-NSL reporter. In the circScreen reporter, the target sequences of the upstream and downstream introns were UCGACGUA and CGAUGUCU respectively. To recognize the endogenous circularized exon, AUGACCGC and UUAGUGCC were selected as ECRR_10720_ upstream/downstream target sequence; ACACAUCA and AUGCGUAU were chosen as ECRR_BIRC6_ upstream and downstream target sequences. The circGFP-NSL or circGFP-SL reporters were constructed as described previously (10). The circScreen reporter was kindly provided by Gregory J. Goodall (Centre for Cancer Biology, SA Pathology and University of South Australia, Adelaide, SA 5000, Australia). See Supplemental information for the sequences of all primers used in our study.

### Cell culture and transfection

HEK293T and UT-HeLa cells were cultured in Dulbecco's modified Eagle's medium (DMEM; with the glucose concentration of 25 mM) supplemented with 10% fetal bovine serum (FBS) at 37 °C with 5% CO_2_. H1299 cells were grown in RPMI-1640 medium supplemented with 10% fetal bovine serum in the presence of 5% CO_2_. Lipofectamine plus (SAGECREATION, China) and Opti-MEM I (Invitrogen) were used for transient transfection according to the manufacturer's protocol. To examine the production of circRNAs from exogenously expressed reporters, 0.5 μg minigenes were transiently transfected into HEK293T cells at 60-70% confluency in 12-well plates. After 36 h, cells were harvested for further analysis. To evaluate the effect of ECRRs on circular RNA generation, 0.12 μg of circ-GFP-reporters were co-transfected with 0.6 μg of ECRRs for each well, and cells were cultured for 48 h or in a time-course and harvested for RNA or protein isolation. To exclude the possibility that the GFP protein could be translated from the linear precursor of circGFP, we digested the reporter plasmid with restriction enzymes that made a single cut or double cuts on the backbone (*ApaL* I or/and *Mlu* I). After gel-purification, 1 μg of the linearized plasmids (*ApaL* I or/and *Mlu* I) were transfected into 293T cells in 12-well plates to examine circGFP expression after 36 h. For further analysis, 0.3 μg of circScreen reporters were co-transfected with 0. 6 μg of ECRRs. The transfected cells were harvested after 48 h. In dose-dependent assays, various ECRRs were transfected as indicated amounts in Figure 3-4. Validation of ECRR function in endogenous circular RNA was confirmed by lentivirus transduction. Briefly, 2 μg of lenti-ECRR vector, 1.5 μg of psPAX2 and 0.5 μg of pMD2.G were co-transfected into 293T cells using Lipofectamine plus (SAGECREATION, China). The culture supernatants were collected at 48 h after transfection and filtered using a 0.45 μM filter. 293T, UT-Hela and H1299 cells were transduced with lentiviral supernatant containing 8 μg/ml Polybrene for 48 h, followed by 2 μg/ml puromycin selection for at least 3 days. Then the ECRR stably expressed cells were seeded into 12-well plates until 40-50% confluency, and subsequently transiently transfected with 50 nM circ10720 or circBIRC6 siRNAs using Lipofectamine 3000 (Catalog No., Invitrogen, USA) according to the manufacturer's protocol, followed by collection cells after 36 h.

### RNA purification and semi-quantitative RT-PCR

Total RNAs was extracted by TRIzol reagent (Invitrogen) according to the manufacturer's protocol, and subsequently treated with DNase I (Promega) at 37 ºC for 30 minutes, followed by 95 ºC for in-activation of DNase I. To digest linear RNA, 1 μg of total RNAs from 293T cells was subjected to 4 U RNase R (Geneseed) treatment in 20 μl reactions at 37 ºC for 20 minutes, followed by RNase R heat inactivation at 70 ºC for 10 min. 1 μg of RNA was applied to synthesize cDNA using Reverse Transcriptase (Takara) according to the manufacturer's instructions. The RT products were then diluted 1:20 used for PCR amplification. Semi-quantitative PCR was performed by incubation at 94 °C for 5 min followed by 25 cycles of 94 °C for 30 s, 61 °C for 30 s, and 72 °C for 10 s on a T100 PCR Thermal Cycler (Bio-Rad). RT-PCR products were then separated on 2.5% agarose gels running with 1× TAE buffer, and scanned with a scanner (Tanon, 2500R). The resulting bands intensity was quantified by Image J software. Real-time PCR was performed using Premix Pro Taq HS qPCR kit (AG11701). ΔΔCt (Cycle threshold) was calculated using housekeeping gene GAPDH. Statistical analysis was performed using a Student's t-test. The primers used in the semi-quantitative PCR and qPCR were listed in Table S1.

### Immunoblotting

Cells were washed twice with cold PBS, and subsequently lysed in RIPA buffer mixed with 1 mM phenylmethyl sulfonyl fluoride (PMSF) and phosphatase inhibitor cocktail (Biomake) for 30 min. Lysates were then centrifuged at 14,000 rpm for 10 min and quantified by the Bradford Protein Assay (Thermo Fisher). Equal amounts of protein samples were separated by SDS-PAGE gel and then transferred to a nitrocellulose membrane (Millipore, Merck, Shanghai, China). Subsequently, membranes were blocked in 5% milk (BD) for 1 h at room temperature (RT) and then incubated with anti-flag (1804, sigma), GFP (11814460001, Roche), HA (H6908, sigma), CUL2 (ABclonal A5308), BIRC6 (Cell Signaling) GAPDH (Cell Signaling) primary antibodies at 4 °C overnight. The membranes were then washed three times using PBS-T, and incubated with peroxidase-conjugated secondary antibodies (1:5000, Cell Signaling) for 1 hour and detected with enhanced chemiluminescence (Tanon).

### Immunoprecipitation analysis

In 60 mm dishes, 70-90% confluent HEK293T cells overexpressing the indicated proteins with FLAG-tag or HA-tag were rinsed with cold PBS before being lysed with cold lysis buffer (50 mM Tris HCl, pH 7.4, with 150 mM NaCl, 1 mM EDTA, 1% TRITONÒ X-100 and protease inhibitor cocktail). The cell lysates were then incubated for 30 minutes on ice on a shaker and centrifuged for 15 min at 12,000 × g at 4 °C. Following the incubation of supernatant with ANTI-FLAG M2 Affinity Gel (sigma, Catalog Number A2220) and rotation overnight at 4 °C, the IP samples were washed three times with wash buffer (50 mM Tris HCl, pH 7.4, with 150 mM NaCl, 10% Glycerol and 0.3% TRITONÒ X-100) and then 100 μg/ml 1xFLAG Peptide was used to elute FLAG fusion proteins from the ANTI-FLAG M2 affinity resins followed by a 15min centrifugation at 10,000 × g at 4 °C. The supernatant was transferred and then boiled in SDS-PAGE loading buffer for western blot analysis.

### Flow cytometry

After transfected with GFP reporters for 36 h, 293T cells were collected and washed twice with cold PBS. Cells were then resuspended in 500 μl PBS with 1% FBS, and analyzed using a FACScan cytometer (Beckon Dickinson, Oxford, UK).

### Northern blotting

CircRNA northern blot assay was basically performed according to the standard method provided by DIG Northern Starter Kit (Roche, Cat. No. 12039672910). 1 μg of total RNA was extracted from 293T cells through Trizol-based method (Life Technologies) and separated by 2% gels with formaldehyde. Gels with total RNA were blotted to a nylon membrane (Solarbio) by capillary transfer overnight. Total RNA on nylon membrane was fixed by baking at 120 °C for 0.5 h. After prehybridizing membrane with DIG Easy Hyb at 68 °C for 30 min, the membranes were hybridized with DIG-labelled probes at 68 °C overnight. The membranes were washed with 2✕ SSC, 0.1✕ SSC, wash buffer, blocking buffer and antibody buffer. Then membranes were exposed to imaging device for 5-20 mins. Data was analyzed using Image Lab software (Bio-Rad).

### Statistics

Quantitative analysis of immunoblotting and RT-PCR was performed using ImageJ software. Differences between experimental groups were evaluated by two-tailed Student's t-test using GraphPad Prism 7 software package to analyze the expression changes and p < 0.05 was considered significant. At least three independent experiments were performed for each dataset, and expressed as the mean ± standard deviation (s.d.). *p < 0.05, **p < 0.01, ***p < 0.001.

## Results

### Design principles of ECRR

**Figure 1:**
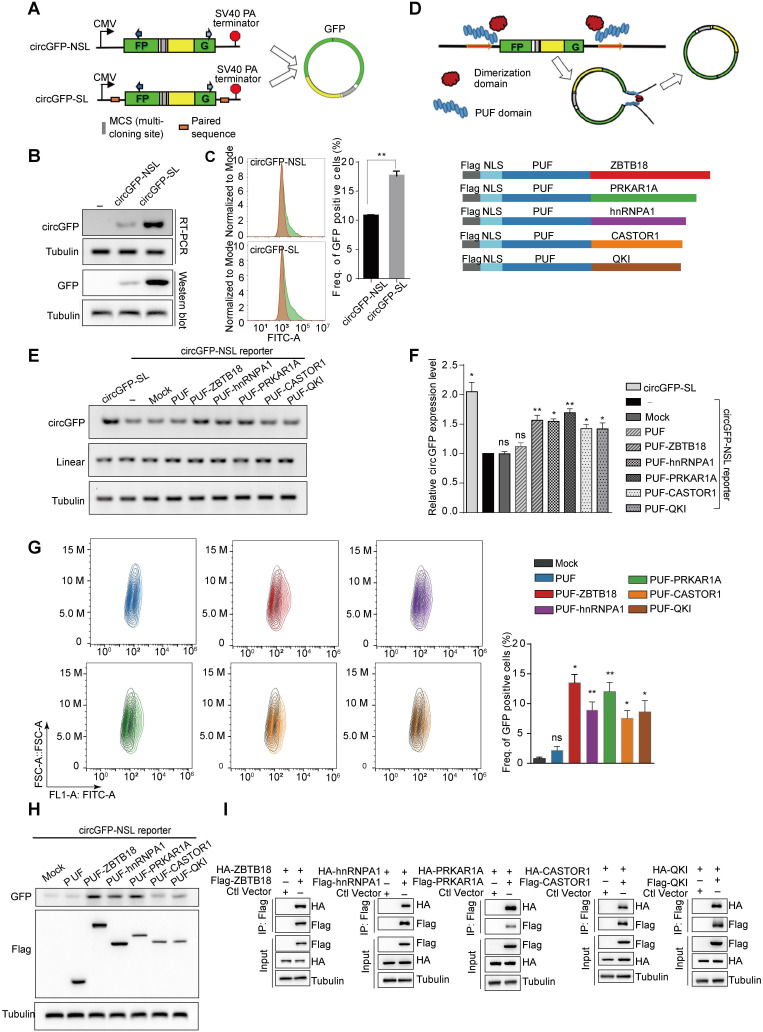
** Designed Engineering Circular RNA Regulators (ECRRs) promote the production of circGFP. (A)** Schematic diagram of circular RNA minigene reporters with the split GFP in a reverse order. The transcription of reporter is driven by a CMV promoter and terminated by SV40 polyadenylation signal. CircGFP-SL contains the inverted complementary sequences in the flanking introns that can form a hairpin structure. CircGFP-NSL does not include any paired sequences in the flanking introns. **(B-C)** The production of circGFP from the minigene reporters. The level of circGFPs generated from the reporters was determined by RT-PCR. The GFP protein level was also examined with a western blot assay (B) and flow cytometry assay (C). **(D)** The schematic diagram of ECRRs. Grey shape indicates the FLAG tag, light and deep blue shape respectively represents the NLS sequence and PUF domain, which is fused to the dimerization protein (ZBTB18, PRKAR1A, hnRNP A1, CASTOR1, and Quaking) in five distinct colors. **(E-F)** ECRRs promote the production of circGFP. The regulatory effects of ECRR (PUF-ZBTB18), ECRR (PUF-hnRNP A1), ECRR(PUF-PRKAR1A), ECRR (PUF-CASTOR), and ECRR (PUF-QKI) on the biogenesis of circGFP were determined using RT-PCR assay. The representative gel was shown in (E). The densities of signals were quantified by Image J and three experiments were carried out with fold change (mean +/- SD) of circGFP expression in each group relative to (-) group plotted in (F). (-) represents the group only transfected with circGFP-NSL reporter. **(G)** The effects of different ECRRs on circGFP production were further examined with flow cytometry assay. Three experiments were conducted and the frequency of GFP positive cells were plotted with mean +/- SD. * indicates p < 0.05, ** indicates p < 0.01. **(H)** The effects of ECRRs on the production of circGFP were determined with a western blot assay. **(I)** The dimerization ability of PRKAR1A, CASTOR, ZBTB18, hnRNPA1, and QKI were examined with a co-immunoprecipitation assay.

To develop and evaluate the novel circRNA modulation tool, we used the circGFP reporters that were constructed as described previously 17. Briefly, circGFP reporters contain two reversed GFP fragments, which could generate a circRNA through back-splicing, thereby producing a functional GFP protein (Figure 1A). CircGFP-SL is similar to the commonly used circRNA expression vector and contains the inverted complementary sequences in the flanking introns that brings splice sites into close proximity, whereas circGFP-NLS didn't include any paired sequence (Figure 1A). We transfected the circGFP reporters into 293T cells respectively and assayed for the production of circRNAs using semi-quantitative RT-PCR one day after transfection. As expected, we detected the production of circRNAs from both circGFP reporters, however, the circRNA biogenesis from circGFP-SL was more efficient than that from circGFP-NSL (Figure 1B). The circRNA was further validated with Sanger sequencing (Figure S1A). In addition, GFP protein could be translated from circGFP as detected by both western blot and flow cytometry assays (Figure 1B-C). To rule out the possibility that transcription from the reporter might bypass the poly-A site at the end to produce a long RNA product from the entire plasmid that contains concatenated GFP fragments, thereby forming a linear RNA containing intact ORF to drive GFP translation, we digested the reporter plasmid with restriction enzymes that make a single cut or double cuts on the backbone (*ApaL* I or/and *Mlu* I). The linearized DNA was gel-purified and transfected into 293T cells. In this case, the transcription can't go around the entire plasmid. As expected, we still detected production of GFP protein (Figure S1B-C), supporting that GFP proteins are indeed translated from circRNAs.

CircRNAs are mainly derived from pre-mRNA back-splicing, and looping the flanking sequence can bring the downstream splice-donor site and upstream splice-acceptor site into close proximity. Certain *trans*-regulatory RNA binding proteins are reported to mediate in closing 3′ and 5′ splice site and promote the production of circRNAs. Particularly, it has been previously reported that the RNA binding protein QKI could bind to the introns flanking circRNA-forming exons (51-342bp from the splicing site) to form a dimer and promote circRNA biogenesis 24. In addition, the RNA binding protein MBL could also promote circRNA production by binding to the flanking introns and stimulating intron-intron interactions 19. However, the binding sites of QKI or MBL are required for them to help circRNA biogenesis, which makes it less practical to manipulate the production of any circRNA.

Based on the characteristics of those RNA binding proteins in circRNA regulation, we proposed to develop engineering circRNA regulators (ECRRs) that could bind to pre-mRNAs and form homodimers to promote the generation of circRNAs. Our idea is to develop ECRRs with both RNA binding domains that bind to short RNA elements with moderate affinities, and functional domains that form homodimers to bring the circle-forming exons together (Figure 1D). To this end, we applied the PUF domain of human Pumilio1 as the RNA binding motif, which contains eight PUF repeats that recognize eight consecutive RNA bases with each repeat binding to a single base. Two amino acid side chains in each repeat recognize the Watson-Crick edge of the corresponding base and determine the specificity of that repeat, thus a PUF domain can be modified to specifically bind to most 8-nt RNA sequences 25-27. Meanwhile, we utilized multiple proteins that could form homodimers, including ZBTB18, PRKAR1A, hnRNP A1, CASTOR1, and QKI, as the functional domains. Subsequently, we developed ECRRs by fusing the PUF domain to distinct dimer-forming proteins respectively to test our design concept. A nuclear localization sequence (NLS) was also inserted to direct ECRRs to the nucleus where splicing occurs and a FLAG epitope tag was introduced to facilitate detection (Figure 1D).

We co-transfected these developed ECRRs with the circGFP-NSL reporter into 293T cells respectively, and assayed for the production of circGFP using the RT-PCR approach. Meanwhile, we used circGFP-SL, which contains the inverted complementary sequences as a positive control for overexpressing circRNAs. The PUF domain of ECRRs was designed to specifically recognize the same 8-nt target sequences in both the upstream (345 nt to 3′ splice site) and the downstream (192 nt to 5′ splice site) flanking introns of the circGFP-NSL reporter. The resulting ECRRs indeed significantly promoted the production of circGFP as efficient as circGFP-SL, the circRNA overexpressing vector, compared to the control (-) that was only transfected with circGFP-NSL, while mock control and PUF-domain-only control had no influence on the circGFP biogenesis (Figure 1E-F). Whereas, the linear RNA production was not affected by ECRRs (Figure S1D). Additionally, such increased production of circGFP led by the application of ECRRs was further confirmed with RNase R treatment, which ensured no linear product was detected (Figure S1E-F).

We also measured the GFP protein production using flow cytometry to determine the efficiency of ECRRs on circRNAs generation. About 1% to 2% of green cells were detected in control cells, whereas cells transfected with specific ECRRs demonstrated seven- to fourteen-fold increase of green cells as compared to control (Figure 1G). The expression level of ECRRs and the generated GFP in the transfected cells were examined with a western blot assay (Figure 1H).

In our design principle of ECRRs, we used multiple proteins, which could form homodimers, as the functional domains. We therefore tested whether these proteins could indeed form a homodimer in this scenario. To this end, we transiently transfected 293T cells with Flag-tagged ZBTB18 and HA-tagged ZBTB18 simultaneously, and applied immunoprecipitation (IP) assay to examine whether Flag-ZBTB18 could interact with HA-ZBTB18. As expected, we found that ZBTB18 could indeed form a homodimer (Figure 1I). The dimerization of other proteins, including hnRNP A1, PRKAR1A, QKI, and CASTOR1, were also revealed in a similar Co-IP assay (Figure 1I). Altogether, our data demonstrated that ECRRs could specifically promote the production of circRNAs through forming a homodimer.

### Dimerization domain provides the ECRRs with potently circRNA-promoting activity

We initially utilized proteins that could form homodimers as the functional domain in our developed ECRRs. However, these proteins contain not only dimerization domain but also other functional domains, which may cause the by-effect of resulting ECRRs. Such effects include interfering the mechanism of biological molecules, thus to influence a plurality of biological behaviors of transfected cells. Therefore, we sought to determine whether only the dimerization domain of the protein is capable of working as the functional domain.

**Figure 2:**
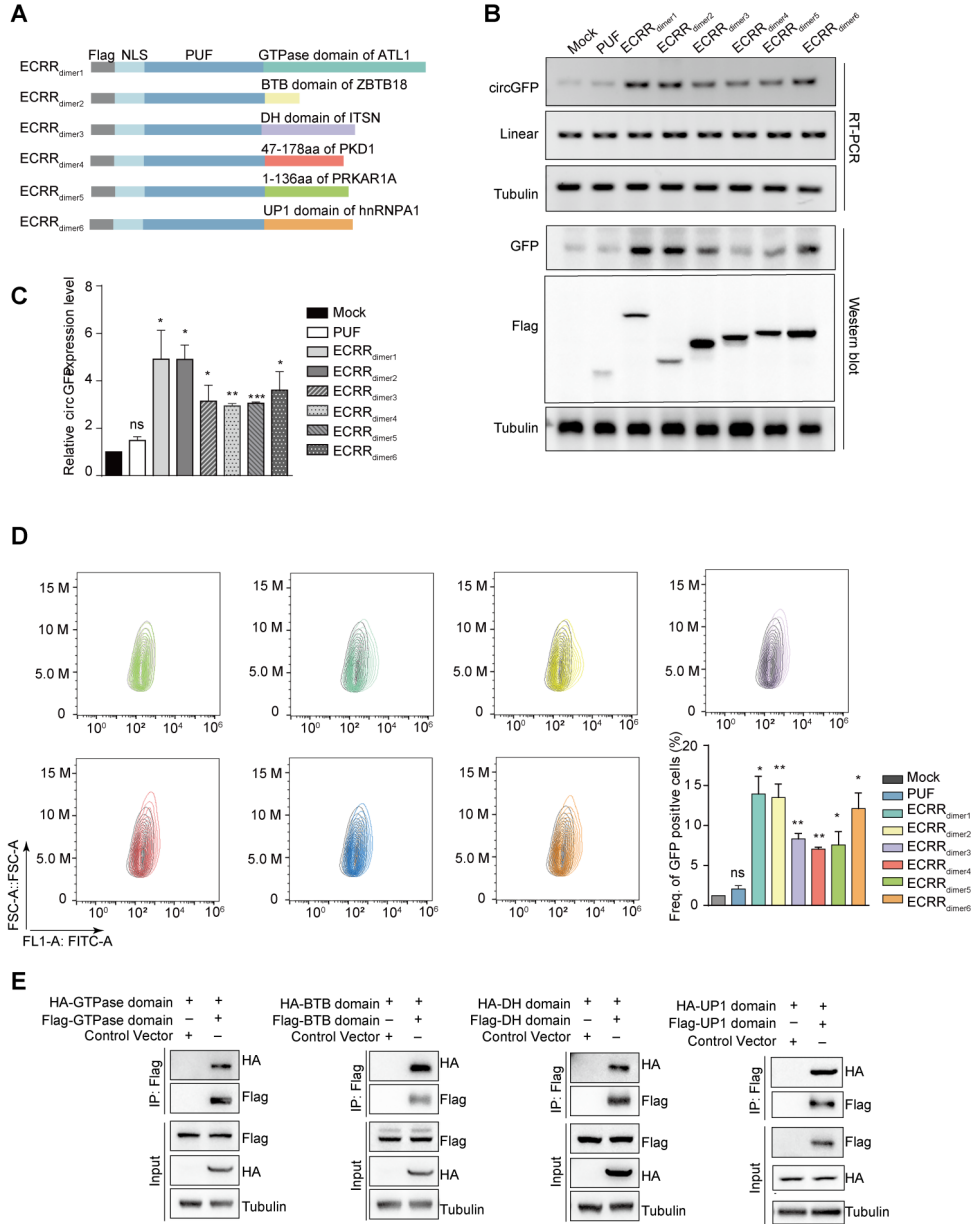
** Dimerization domains are strong enough for ECRRs to promote the circGFP biogenesis. (A)** The schematic diagram of ECRRs that contain only the dimerization domain. (dimer1: GTPase domain of ATL1, dimer2: BTB domain of ZBTB18, dimer3: DH domain of ITSN1, dimer4: 47-178 aa of PKD1, dimer5: 1-136 aa of PRKAR1A, dimer6: UP1 (RRM) domain of hnRNPA1).** (B-C)** The regulatory effects of different ECRRs on the biogenesis of circGFP were determined using RT-PCR and western blot assays. The representative gel was shown in (B). The greyscale values were determined by Image J and three experiments were carried out with mean +/- SD of relative fold change of circGFP expression to mock group plotted in (C). * indicates p < 0.05, ** indicates p < 0.01. **(D)** The effects of ECRRs containing different dimerization domains on circGFP production were further examined with flow cytometry assay. Three experiments were conducted and the frequency of GFP positive cells were plotted with mean +/- SD. * indicates p < 0.05, ** indicates p < 0.01. **(E)** The dimerization of GTPase domain, BTB domain, ITSN domain, PKD domain, PKA domain, and RRM domain was examined with a co-immunoprecipitation assay. The representative gel figures were shown.

We only fused the dimerization domain of proteins, including GTPase domain of ATL1, BTB domain of ZBTB18, DH domain of ITSN1, 47-178 aa of PKD1, 1-136 aa of PRKAR1A, and UP1 (RRM) domain of HNRNPA1, to PUF domain respectively to establish the new ECRR_dimers_ (Figure 2A). Subsequently, we transiently transfected these ECRRs with circGFP-NSL reporter into 293T cells, and examined their functions in regulating circRNA biogenesis. As expected, the newly designed ECRRs could significantly stimulate the biogenesis of circGFP, but not the linear RNA, as compared to the controls (Figure 2B-C and Figure S2A). Importantly, ECRR_dimer1_ (ECRR(PUF-GTPase)) and ECRR_dimer2_ (ECRR(PUF-BTB)) could most efficiently promote the production of circGFP as five times as the control does (Figure 2B-C). Similar results were also obtained in the presence of RNase R treatment (Figure S2B-C). The expression levels of GFP and Flag-tagged ECRRs in the transfected cells were examined with a western blot assay (Figure 2B). Consistently, functions of the new ECRRs, which only contain the dimerization domains, were also validated by using flow cytometry to examine the GFP protein production, revealing that about six to fifteen folds elevation of green signal was obtained in cells with transient transfection of specific ECRRs as compared to cells with control (Figure 2D). In addition, such ECRRs-promoted production of circGFP was also validated with the northern blot assay (Figure S2D). Altogether, our data demonstrated that only the dimerization domain provides the ECRRs with potently circRNA-promoting activity.

To further examine whether only the dimerization domain of those proteins could indeed form homodimers, we transiently transfected 293T cells with Flag-GTPase and HA-GTPase simultaneously, and used immunoprecipitation assay to measure their interactions. As expected, the GTPase domain of ATL1 could form a homodimer in cells as judged by the Co-IP assay (Figure 2E). In addition, the interaction of multiple dimerization domains was also validated, including BTB domain of ZBTB18, DH domain of ITSN, UP1 domain of hnRNPA1 (Figure 2E), 47-178 aa of PKD1, and 1-136 aa of PRKAR1A (Figure S2E). Taken together, our results demonstrated that only the dimerization domain is strong enough for ECRRs to form homodimers, thereby providing nearly the same effect as the entire protein to promote circRNA production.

### ECRRs promote circRNAs biogenesis in time course- and dose-dependent manners

**Figure 3:**
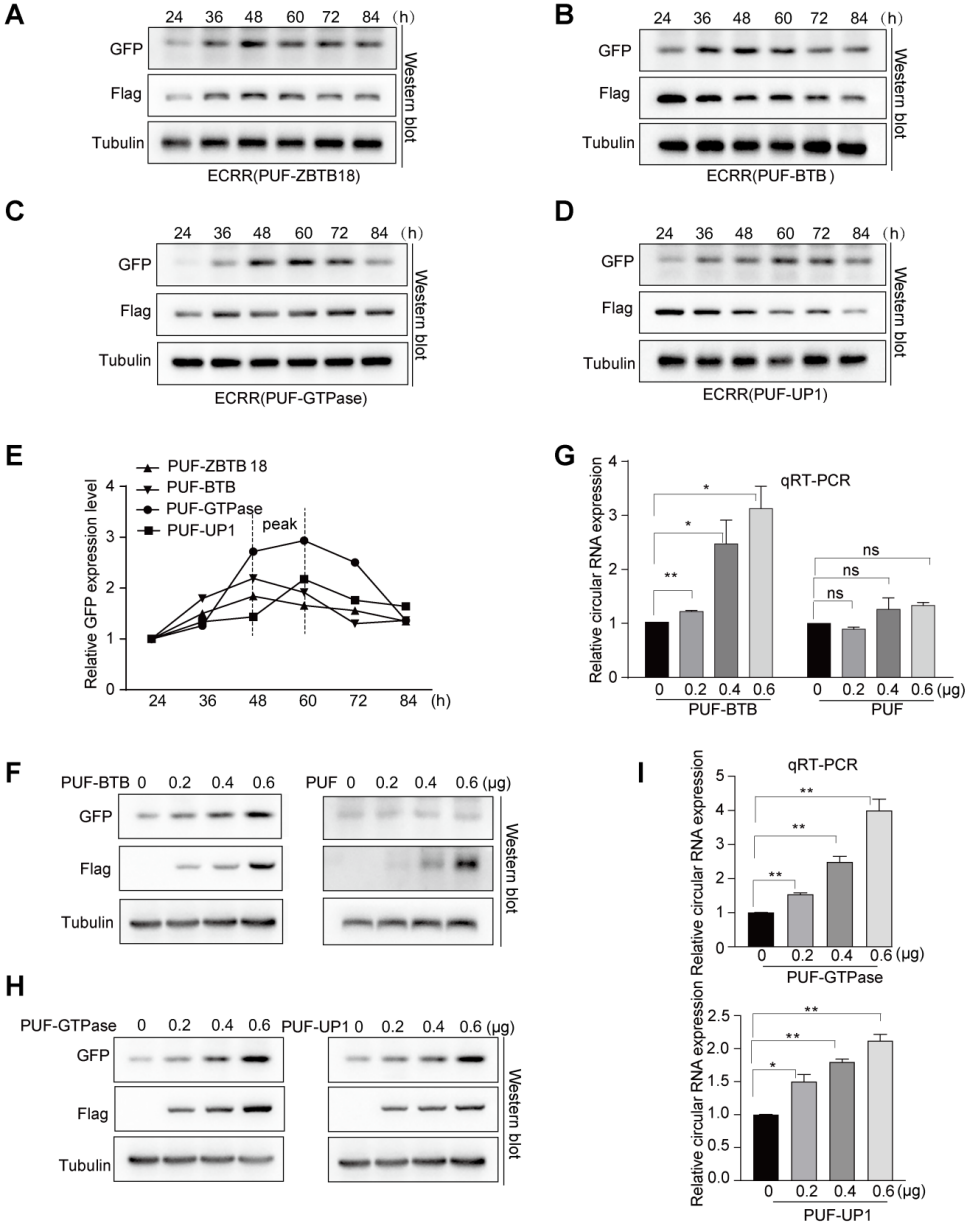
** ECRRs promote the circGFP biogenesis in time-course dependent and dose-dependent manners with designed specificity. (A-D)** The circGFP-NSL reporter was co-transfected with ECRR(PUF-ZBTB18) (A), ECRR(PUF-BTB) (B), ECRR(PUF-GTPase) (C) or ECRR(PUF-UP1) (D), then the transfected cells were collected at 24, 36, 48, 60, 72, and 84 hours after transfection. The protein levels of GFP and Flag were determined with a western blot assay. **(E)** The tendency of GFP expression level of each group in time-course was analyzed by a line chart. **(F-G)** The fixed amount of the circGFP-NSL reporter was co-transfected with increased amounts (0, 0.2, 0.4, 0.6 µg) of ECRR(PUF-BTB) or control ECRR(PUF-only) into 293T cells. The resulting cells were harvested 48 hours after transfection, and the protein levels of GFP and Flag were examined with a western blot assay. The representative figure was shown in (F). The RNA levels of circGFP were determined by qRT-PCR. Data presented as circRNA abundance relative to 0μg ECRR transfected cells, mean ± SEM, n = 3 (G). * indicates p < 0.05, ** indicates p < 0.01. **(H-I)** The fixed amount of the circGFP-NSL reporter was co-transfected with increased amounts (0, 0.2, 0.4, 0.6 µg) of ECRR (PUF-GTPase) or ECRR (PUF-UP1) into 293T cells. The resulting cells were harvested 48 hours after transfection, and the protein levels of GFP and Flag were examined with a western blot assay. The representative figure was shown in (H). The corresponding circGFP RNA abundance were measured by qRT-PCR with data showed as circRNA exression level relative to 0 µg ECRR transfected cells, mean ± SEM, n = 3 (I). * indicates p < 0.05, ** indicates p < 0.01.

We next sought to investigate the efficiency of ECRRs in regulating the production of circRNAs. We chose ECRR(PUF-ZBTB18) that contains PUF domain and the entire ZBTB18 protein, as well as ECRR(PUF-BTB) that includes PUF domain and the BTB domain of ZBTB18 protein, which are the top two ECRRs that could significantly promote the production of circGFP as shown previously (Figures 1E, 1G, 2B, 2D). We transiently co-transfected the circGFP-NSL reporter with ECRR(PUF-ZBTB18) or ECRR(PUF-BTB) into cells and collected the transfected cells at multiple time points, including 24, 36, 48, 60, 72, and 84 hours after transfection. As expected, ECRR(PUF-ZBTB18) and ECRR(PUF-BTB) could promote the generation of circGFP in a time course-dependent manner (Figure 3A-B). In addition, the production of circGFP could also be stimulated in a time course-dependent manner when treated with ECRR(PUF-GTPase), and ECRR(PUF-UP1) (Figure 3C-D). The production of circGFP reached the peak from 48 to 60 hours post-transfection (Figure 3E).

Moreover, we co-transfected a fixed amount of circGFP-NSL reporter with increased doses of ECRR(PUF-BTB). Two days after transfection, we measured the production of circGFPs and revealed that the generation of circGFP was dose-dependently elevated with the increased amounts of ECRR(PUF-BTB) (Figure 3F-G), however, the ECRR(PUF-only) control that could not form a dimer, had no effect on the production of circGFP (Figure 3F-G). Similar results were also obtained when the circGFP reporter was co-transfected with distinct amounts of ECRR(PUF-GTPase) and ECRR(PUF-UP1) (Figure 3H-I). Altogether, our data established the concept that ECRRs could efficiently promote the production of circRNAs with designed specificity.

### ECRR promotes circRNA production in a different circRNA reporter

**Figure 4:**
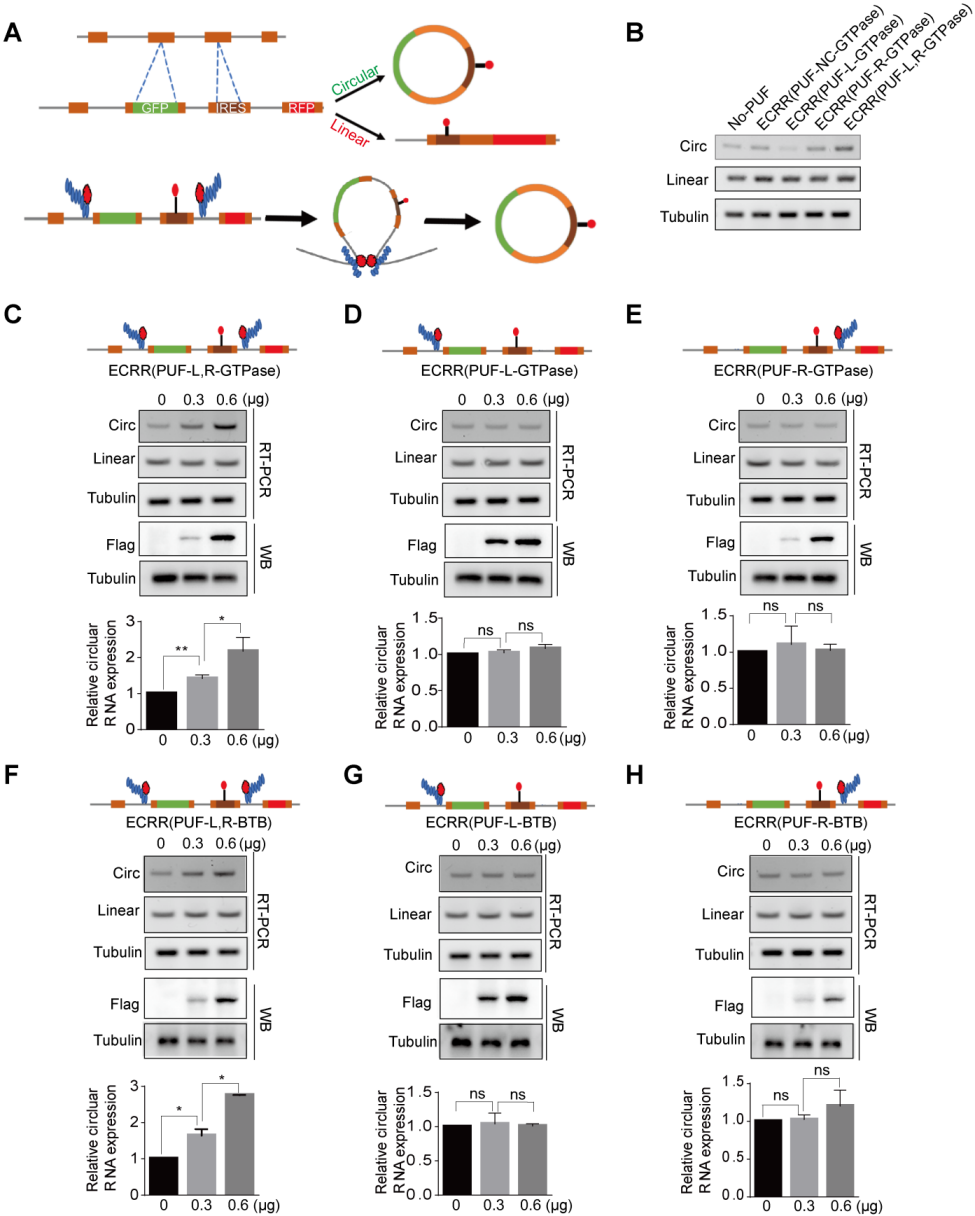
** ECRRs stimulate the circular RNA production of the circScreen minigene reporter. (A)** The schematic diagrams of how ECRRs regulate the circular RNA biogenesis of the circScreen minigene reporter. **(B)** The effects of different ECRRs on the circular RNA production of circScreen reporter were examined with RT-PCR. The representative gel figures were shown. **(C)** The fixed amount of the circScreen reporter was co-transfected with increased amounts of ECRR (PUF-L-GTPase) and ECRR (PUF-R-GTPase), whose PUF domains recognized both the upstream and downstream target sequences simultaneously, into 293T cells. **(D)** The fixed amount of the circScreen reporter was co-transfected with increased amounts of ECRR (PUF-L-GTPase), whose PUF only recognized the upstream target sequence in the reporter, into 293T cells. **(E)** The fixed amount of the circScreen reporter was co-transfected with increased amounts of ECRR (PUF-R-GTPase), whose PUF only recognized the downstream target sequence in the reporter, into 293T cells. For (C-E), the resulting cells were harvested 48 hours after transfection, and total RNAs were isolated for RT-PCR to examine the level of circular and linear RNA. **(F)** The fixed amount of the circScreen reporter was co-transfected with increased amounts of ECRR (PUF-L-BTB) and ECRR (PUF-R-BTB), whose PUF domains recognized both the upstream and downstream target sequences simultaneously, into 293T cells. **(G)** The fixed amount of the circScreen reporter was co-transfected with increased amounts of ECRR (PUF-L-BTB), whose PUF only recognized the upstream target sequence in the reporter, into 293T cells. **(H)** The fixed amount of the circScreen reporter was co-transfected with increased amounts of ECRR (PUF-R-BTB), whose PUF only recognized the downstream target sequence in the reporter, into 293T cells. For panels (F-H), the resulting cells were harvested 48 hours after transfection, and total RNAs were isolated for RT-PCR to examine the level of circular and linear RNA. The representative gel figure was shown. The densities of signals were determined by densitometry and three experiments were carried out with mean +/- SD of relative fold change of circular RNA expression plotted. The protein level of FLAG was also measured with a western blot assay.

We have demonstrated that ECRRs can be used to modulate circRNA production of circGFP-NSL reporter. However, it still remains elusive whether such regulation could be commonly applied to other reporters. We therefore employed another circRNA reporter, circScreen, to examine the promoting activity of ECRRs on circRNA biogenesis. The circScreen reporter was constructed from SMARCA5 as described previously 24. The green signal indicates the circRNAs, whereas the red signal represents the linear RNAs (Figure 4A). We transfected 293T cells with increased amounts of the circScreen reporter. Two days after transfection, we extracted RNAs from transfected cells and assayed for the production of circRNAs and linear RNAs by the RT-PCR assay. Both of the circRNAs and linear RNAs were detected, and the production was elevated with the increased amounts of the reporter (Figure S3A). In addition, the circRNA products were validated with Sanger sequencing (Figure S3B).

We further developed multiple ECRRs to evaluate their effects on the circRNA biogenesis of the circScreen reporter. As a negative control, ECRR(PUF-NC-GTPase), which contained a control PUF domain that does not recognize the sequences in the reporter, did not affect the biogenesis of circRNAs (Figure 4B). Moreover, ECRR(PUF-L-GTPase) and ECRR(PUF-R-GTPase), which only recognized either upstream or downstream target sequence in the circScreen reporter, could not obviously influence the circRNA generation (Figure 4B). However, the simultaneous application of ECRR(PUF-L-GTPase) and ECRR(PUF-R-GTPase), which separately bound two distinct target sequences in the upstream and downstream introns to form a dimer, noticeably promoted the production of circRNAs (Figure 4B).

Furthermore, we measured the regulatory efficiency and specificity of ECRRs on circScreen reporter biogenesis. The simultaneous application of ECRR(PUF-L-GTPase) and ECRR(PUF-R-GTPase) could stimulate the production of circRNAs in a dose-dependent manner, whereas it did not affect the production of linear RNAs (Figure 4C). However, the amount of circRNAs were not evidently influenced with the increased application of ECRR(PUF-L-GTPase) or ECRR(PUF-R-GTPase) separately (Figure 4D-E). Consistently, the simultaneous application ECRR(PUF-L-BTB) and ECRR(PUF-R-BTB) that specifically bound both upstream and downstream introns of the reporter could also promote the production of circScreen accordingly (Figure 4F), but separate application of ECRR(PUF-L-BTB) or ECRR(PUF-R-BTB), which only bound either upstream or downstream introns of the circScreen reporter, had no effect on the circRNA biogenesis (Figure 4G-H). Taken together, our data demonstrated that the engineering circRNA regulators could be applied to promote circRNA biogenesis in distinct reporters, proving that such artificial regulation could be commonly used in exogenously expressed circRNA reporters.

### ECRRs stimulate the biogenesis of endogenous circular RNAs

**Figure 5:**
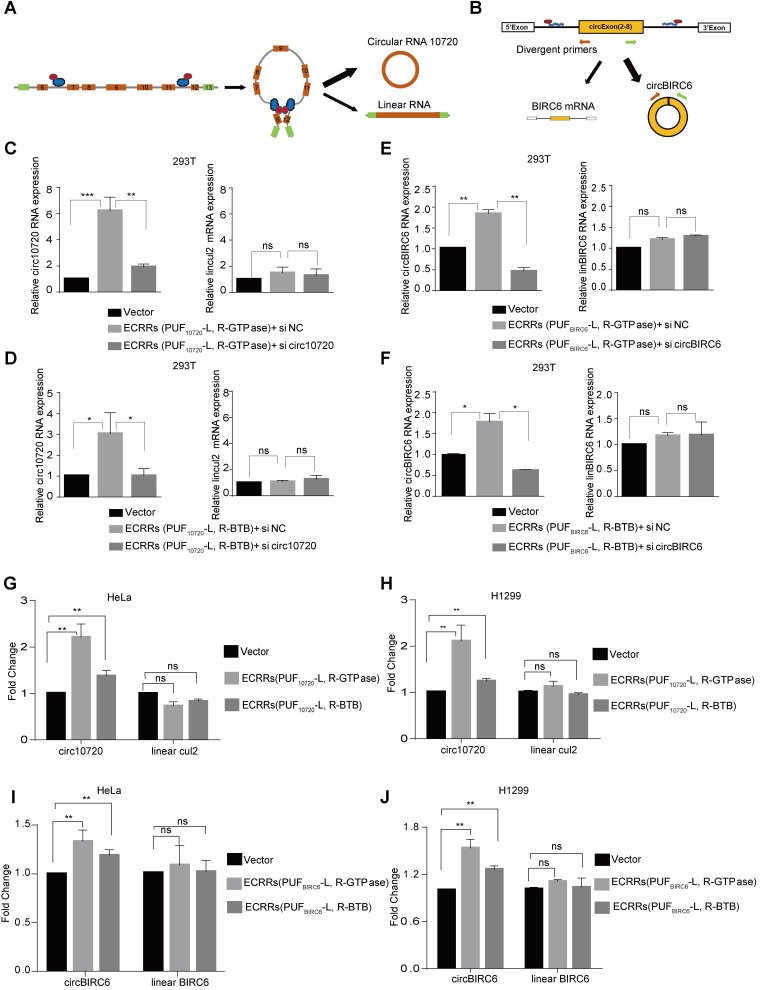
** ECRRs promote the endogenous circular RNAs production. (A-B)** The schematic diagrams of how ECRRs modulate the production of the endogenous circular RNA circ10720 (A) or circBIRC6 (B). **(C)** ECRR (PUF_10720_-L-GTPase) and ECRR (PUF_10720_-R-GTPase) were stably transfected into 293T cells. The resulting cells were transiently transfected with circ10720 siRNA or control siRNA. After forty-eight hours, total RNAs were isolated from the transfected cells, and qRT-PCR assay was applied to examine the levels of circ10720 and linear RNA (CUL2 E4-E5). Three experiments were carried out with mean +/- SD of relative fold change of circ10720 and linear Cul2 expression plotted. **(D)** ECRR (PUF_10720_-L-BTB) and ECRR(PUF_10720_-R-BTB) were stably transfected into 293T cells. The resulting cells were transiently transfected with circ10720 siRNA or control siRNA. After forty-eight hours, total RNAs were isolated from the transfected cells, and qRT-PCR assay was applied to examine the levels of circ10720 and linear RNA (CUL2 E4-E5). Three experiments were carried out with mean +/- SD of relative fold change of circ10720 and linear Cul2 expression plotted. **(E)** ECRR (PUF_BIRC6_-L-GTPase) and ECRR (PUF_BIRC6_-R-GTPase) were stably transfected into 293T cells. The resulting cells were transiently transfected with circBIRC6 siRNA or control siRNA. After forty-eight hours, total RNAs were isolated from the transfected cells, and qRT-PCR assay was applied to examine the levels of circBIRC6 and linear BIRC6 RNA. Three experiments were carried out with mean +/- SD of relative fold change of circBIRC6 and linear BIRC6 expression plotted. **(F)** ECRR (PUF_BIRC6_-L-BTB) and ECRR (PUF_BIRC6_-R-BTB) were stably transfected into 293T cells. The resulting cells were transiently transfected with circBIRC6 siRNA or control siRNA. After forty-eight hours, total RNAs were isolated from the transfected cells, and qRT-PCR assay was applied to examine the levels of circBIRC6 and linear BIRC6 RNA. Three experiments were carried out with mean +/- SD of relative fold change of circBIRC6 and linear BIRC6 expression plotted. For panels C to J, * indicates p < 0.05, ** indicates p < 0.01, *** indicates p < 0.001. **(G-H)** ECRRs (PUF_10720_-L, R-GTPase) or ECRRs (PUF_10720_-L, R-BTB) were stably transfected into HeLa (G) or H1299 (H) cells. Total RNAs were isolated from the transfected cells, and qRT-PCR assay was applied to examine the levels of circ10720 and linear RNA (CUL2 E4-E5). Three experiments were carried out with mean +/- SD of relative fold change of circ10720 and linear Cul2 expression plotted. **(I-J)** ECRRs (PUF_BIRC6_-L, R-GTPase) or ECRRs (PUF_BIRC6_-L, R-BTB) were stably transfected into HeLa (I) or H1299 (J) cells. Total RNAs were isolated from the transfected cells, and qRT-PCR assay was applied to examine the levels of circBIRC6 and linear BIRC6 RNA. Three experiments were carried out with mean +/- SD of relative fold change of circBIRC6 and linear BIRC6 expression plotted. For panels C to J, * indicates p < 0.05, ** indicates p < 0.01, *** indicates p < 0.001.

We subsequently applied ECRRs to promote the production of two endogenous circRNAs. One was circ10720, an endogenous Cul2 circRNA, which is generated from exon 7 to exon 11 (Figure 5A) 28. The other endogenous circRNA was circBIRC6 that is produced from exon 2 to exon 8 (Figure 5B) 29.

To minimize the off-target effect of ECRRs, we developed ECRR(PUF_10720_-L-GTPase) and ECRR(PUF_10720_-R-GTPase) to modulate the circ10720 biogenesis, whose PUF domains recognized two distinct target sequences located in intron 6 and intron 11 of Cul2 pre-mRNAs respectively. The specificity of these ECRRs on endogenous circRNA biogenesis was examined. Firstly, ECRR(PUF_10720_-L-GTPase) and ECRR(PUF_10720_-R-GTPase) were stably transfected into cells, and the endogenous circ10720 was subsequently depleted with specific siRNAs. As expected, the simultaneous application of ECRR(PUF_10720_-L-GTPase) and ECRR(PUF_10720_-R-GTPase) promoted production of endogenous circ10720 could be specifically inhibited by circ10720 siRNAs, suggesting that our designed ECRRs could promote the endogenous circRNA biogenesis with specificity (Figure 5C). However, the Cul2 linear RNA was not affected (Figure 5C). Similar results were also obtained with the application of ECRR(PUF_10720_-L-BTB) and ECRR(PUF_10720_-R-BTB) (Figure 5D). Additionally, the elevated circ10720 biogenesis led by ECRR(PUF_10720_-L, R-GTPase) or ECRR(PUF_10720_-L,R-BTB) was also verified with a northern blot assay (Figure S4A). The sequences of endogenous circ10720 were further validated with Sanger sequencing, and the expression levels of ECRRs and Cul2 protein were measured with a western blot assay (Figure S4B-C). In addition, ECRR(PUF_BIRC6_-L, R-GTPase) or ECRR(PUF_BIRC6_-L, R-BTB) were developed to recognize two different target sequences located in the flanking intron of circBIRC6 respectively, thereby specifically stimulating the generation of circBIRC6 as judged by qRT-PCR and northern blot assays (Figure 5E-F and Figure S4D-F).

ECRRs were also utilized to promote the production of endogenous circ10720 and circBIRC6 in HeLa and H1299 cells. Consistently, the application of ECRRs(PUF_10720_-L, R-GTPase) or ECRRs(PUF_10720_-L, R-BTB) significantly stimulated the production of endogenous circ10720 in both HeLa and H1299 cells as determined by qRT-PCR (Figure 5G-H and Figure S4G). However, the expression levels of Cul2 linear RNA were not influenced by the specific ECRRs (Figure 5G-H and Figure S4G). Similarly, ECRRs(PUF_BIRC6_-L, R-GTPase) or ECRRs(PUF_BIRC6_-L, R-BTB) could also promote the production of the endogenous circBIRC6, but not the BIRC6 linear RNA in HeLa and H1299 cells (Figure 5I-J and Figure S4H).

Collectively, our results proved that the application of ECRRs could not only stimulate the biogenesis of exogenous circRNA reporters, but also promote the production of endogenous circRNA. Most importantly, such regulation was in a sequence-specific manner.

## Discussion

A large number of circRNAs have been discovered in the mammalian transcriptome with high abundance 4-7, but only a minority of circRNAs have been reported to have important biological functions. Due to deficient technology and experimental methods, especially the limitations of overexpression strategies *in vivo*, the functions of most circRNAs are still unknown. Hence engineering circRNA regulators to specifically promote the production of circRNAs should be able to offer tremendous promise for both basic and translational research. Remarkably, either some *trans*-regulatory RBPs or flanking inverted repeat elements, which are involved in bringing the downstream splice-donor site and the upstream splice-acceptor site into close proximity, can promote the production of circRNAs through back splicing. Following this principle, we developed an efficient tool ECRR that is constructed by combining PUF domain, a sequence-specific RNA-binding motif that could bind to any position in the pre-mRNAs, and a functional domain, which is responsible for bringing splice sites closer through dimerization.

To verify our hypothesis, we first utilized multiple proteins, including ZBTB18, PRKAR1A, hnRNP A1, Quaking, and CASTOR1 that could form dimerization, as the functional domains. Several proteins (e.g., hnRNP A1 and QKI) are RNA binding proteins, which have been previously demonstrated to participate in the biogenesis of circRNAs 30, thus we could not exclude the regulatory effects of RBP-containing ECRRs from the direct binding of RBPs to the circRNAs. Meanwhile, the full-length entire proteins may disturb biological behavior of transfected cells. To rule out these possibilities, we expanded our functional domains to a panel of protein domains that could form homodimers but not bind to RNA, such as the GTPase domain of ATL1 protein and the BTB domain of ZBTB18 protein. ATL1 is a membrane-fusing GTPase, whose function is to tether membranes through formation of *trans*-homooligomers 31. Most importantly, ATL1 is not an RNA binding protein, and the function of the GTPase domain of ATL1 is merely to form dimerization. Similarly, the BTB domain of ZBTB18 could form a homodimer as well. Therefore, the application of the GTPase domain and BTB domain clarified that the function of ECRRs only depends on the formation of dimerization to bring the circle-forming exons into close proximity to stimulate the production of circRNAs.

Although the dimerization domains we used in ECRRs could promote circRNA biogenesis, their regulatory roles in the production of circRNAs are quite different. This might be because the structures of proteins vary a lot, thereby leading to distinct affinities when they form homodimers. Moreover, the successful generation of ECRRs proves our design principle is correct. Thus, the application of ECRRs provide a novel approach to study the biogenesis of circRNAs by specifically recruiting different dimerization protein domains to certain pre-mRNA regions.

Currently, the most popular strategy for overexpressing circRNAs is to transfect cells by using circRNA-producing plasmid in which the circularizing sequence is flanked by canonical splice sites and inverted complementary sequences. For example, the production of circRNA ciRS-7 was promoted through a vector-based system that contained an inserted ciRS-7 locus (including 1 kb upstream and 200 bp downstream). Importantly, partial upstream sequence was inverted and inserted at the downstream locus, thereby significantly increasing ciRS-7 outputs and reducing additional by-product 8. In addition, the biogenesis of circAAC1 could be promoted by inserting circACC1 sequences that was flanked by canonical AG/GT splice site and two tandem reverse complementary intron sequences in the expression vector 32. However, this strategy that relies on foreign circRNA-producing plasmid usually results in the random insertion of the circRNA expression locus. Meanwhile, linear transcripts and circular concatemers are co-produced, which could interfere with the estimate for circRNA function. Another commonly used approach to promote circRNA expression is to overexpress specific RBPs, which can bind to the exact site of flanking introns to promote circularization. For instance, QKI could bind to its binding sites in the introns flanking the circRNA-forming exons of SMARCA5, and promote circRNA biogenesis through forming a homodimer to bring circRNA splice sites into close proximity 24. In addition, circMbl biogenesis could be strongly and specifically promoted by muscleblind (MBNL1), which binds to its flanking introns that contain conserved muscleblind binding sites 19. But synthetic QKI and MBNL1 binding sites are needed to be inserted in the introns, which makes it less practical to largely apply this method to improve circRNA expression level without genome modification *in vivo*. Compared with these strategies, the ECRRs we report here can promote circRNA production precisely from genome locus without introducing any linear transcripts *in vivo*. On the other hand, ECRRs can recognize the pre-mRNAs in a natural context without introducing foreign binding sites to the genome. Moreover, the PUF RNA-binding domain of our ECRRs specifically recognizes an 8-nt target sequence, which provides target discriminatory power similar to that of microRNAs that recognize targets mostly by a 7-nt seed match, however, the PUF domain might still have the off-target effect. Therefore, to largely minimize the off-target effects, we applied two ECRRs to simultaneously recognize two distinct target sequences located at both the upstream and downstream introns and form the homodimers, which improves the binding specificity of ECRRs from recognizing an 8-nt to 16-nt target sequence. Collectively, ECRRs are more advantageous for *in vivo* applications.

circRNAs have been demonstrated to play vital roles in a variety of human diseases, including cancer and cardiac vascular disease. For example, circFoxo3 triggers stress-induced apoptosis and inhibits the growth of tumor xenografts 33. In addition, circCcnb1 inhibits the mutant p53-induced enhanced breast cancer progression 34. Moreover, Foxo3 circRNA promotes cardiac senescence by modulating multiple factors associated with stress and senescence responses 11. Therefore, the development of ECRRs also offers a new strategy to regulate circRNA biogenesis, potentially resulting in novel therapeutics for multiple diseases treatment. By optimizing different combinations of ECRR modules, such approach will provide a fine-tuned adjustment of circRNA production. Additionally, ECRRs are capable of stably expressing *in vivo* using delivery tools, thereby making it possible for gene therapy of certain disease.

## Supplementary Material

Supplementary figures and tables.
