# Supplementary material for: Engineering circular RNA regulators to specifically promote circular RNA production
Source: Theranostics. 2021 May 24;11(15):7322–36. doi: 10.7150/thno.56990 (PMC8210604; doi:10.7150/thno.56990)
Supplement: Supplementary file 1 — Supplementary figures and tables. [file thnov11p7322s1.pdf]

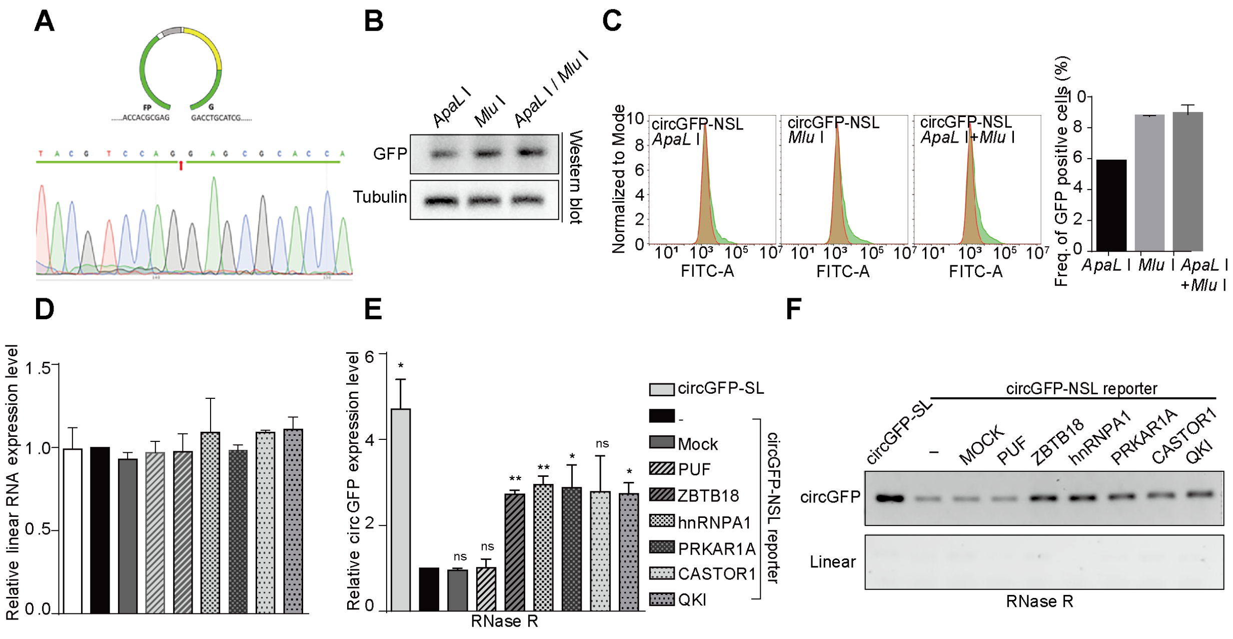

Supplementary Figure S1. ECRRs promote the production of circGFP. **(A)** The junction sequences of circGFP were validated with Sanger sequencing. **(B-C)** The circGFP reporter vector has single *ApaL* I and *Mlu* I enzyme sites in the plasmid backbone. The circGFP vector was linearized with *ApaL* I, *Mlu* I, or *ApaL* I/*Mlu* I digestion, and the resulting linearized DNA was transiently transfected into 293T cells to examine GFP expression at 36 h after transfection. The GFP protein was detected with western blot assay (B) and flow cytometry assay (C). **(D)** The gray-scale value of straps densities was quantified by Image J to detect linear RNA abundance from circGFP reporter in Fig1E. Three experiments were carried out with mean +/- S D plotted in each group. **(E-F)** The regulatory effects of different ECRRs on the biogenesis of circGFP in the presence of RNase R were determined using RT-PCR assay. The densities of signals were determined by Image J and three experiments were carried out with mean +/- SD of relative fold change of circGFP expression plotted in (E). The representative gel was shown in (F). * indicates $p < 0.05$, ** indicates $p < 0.01$.

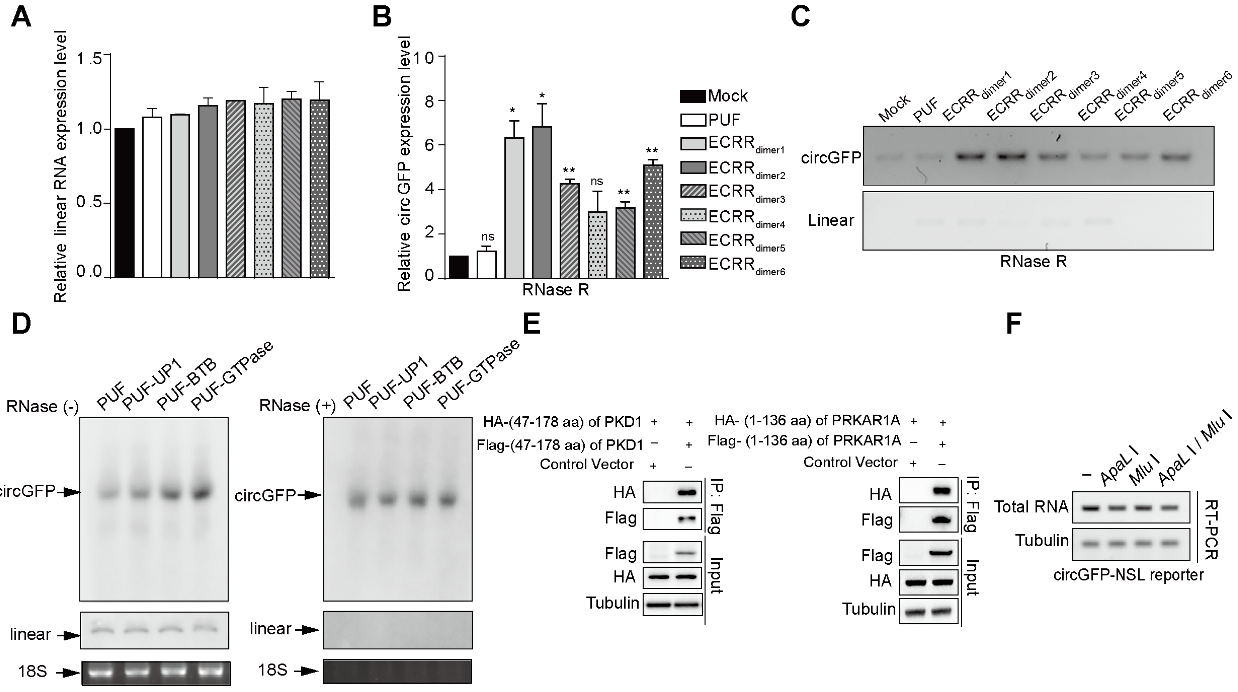

Supplementary Figure S2. Dimerization domain containing ECRRs stimulate the generation of circGFP. **(A)** The densities of RT-PCR bands were quantified by Image J to detect linear RNA abundance from circGFP reporter in Fig2B. Three experiments were carried out with mean +/- SD plotted in each group. **(B-C)** The regulatory effects of different ECRRs containing GTPase domain of ATL1, BTB domain of ZBTB18, DH domain of ITSN1, 47-178 aa of PKD1, 1-136 aa of PRKAR1A, and RRM domain of HNRNPA1 in the presence of RNase R on the biogenesis of circGFP were determined using RT-PCR assay. The densities of signals were determined by Image J and three experiments were carried out with mean +/- SD of relative fold change of circGFP expression plotted in (B). The representative gel was shown in (C). * indicates $p < 0.05$, ** indicates $p < 0.01$. **(D)** The circGFP-NSL reporter was transiently co-transfected with different ECRRs or control vector. The production of circGFP and linear RNA was examined with the northern blot assay in the absence of presence of RNase R treatment. **(E)** The dimerization ability of 47-178 aa of PKD1, 1-136 aa of PRKAR1A were examined with a co-

immunoprecipitation assay. **(F)** 1 μg of the linearized plasmids (*ApaL* I or/and *Mlu* I) were transfected into 293T cells in 12-well plates to examine circGFP expression. To determine the circular and linear transfection efficiency, RNAs were isolated from the transfected cells to measure the efficiency by RT-PCR.

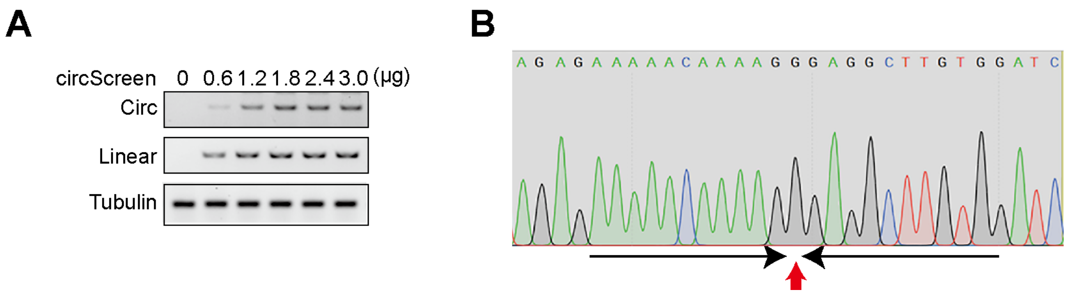

Supplementary Figure S3. ECRRs function in another circular RNA reporter. **(A)** The production of circular RNA and linear RNA of circScreen reporter was analyzed with the RT-PCR approach. The representative gels were shown. **(B)** The junction sequence of the circScreen reporter was verified with Sanger sequencing.

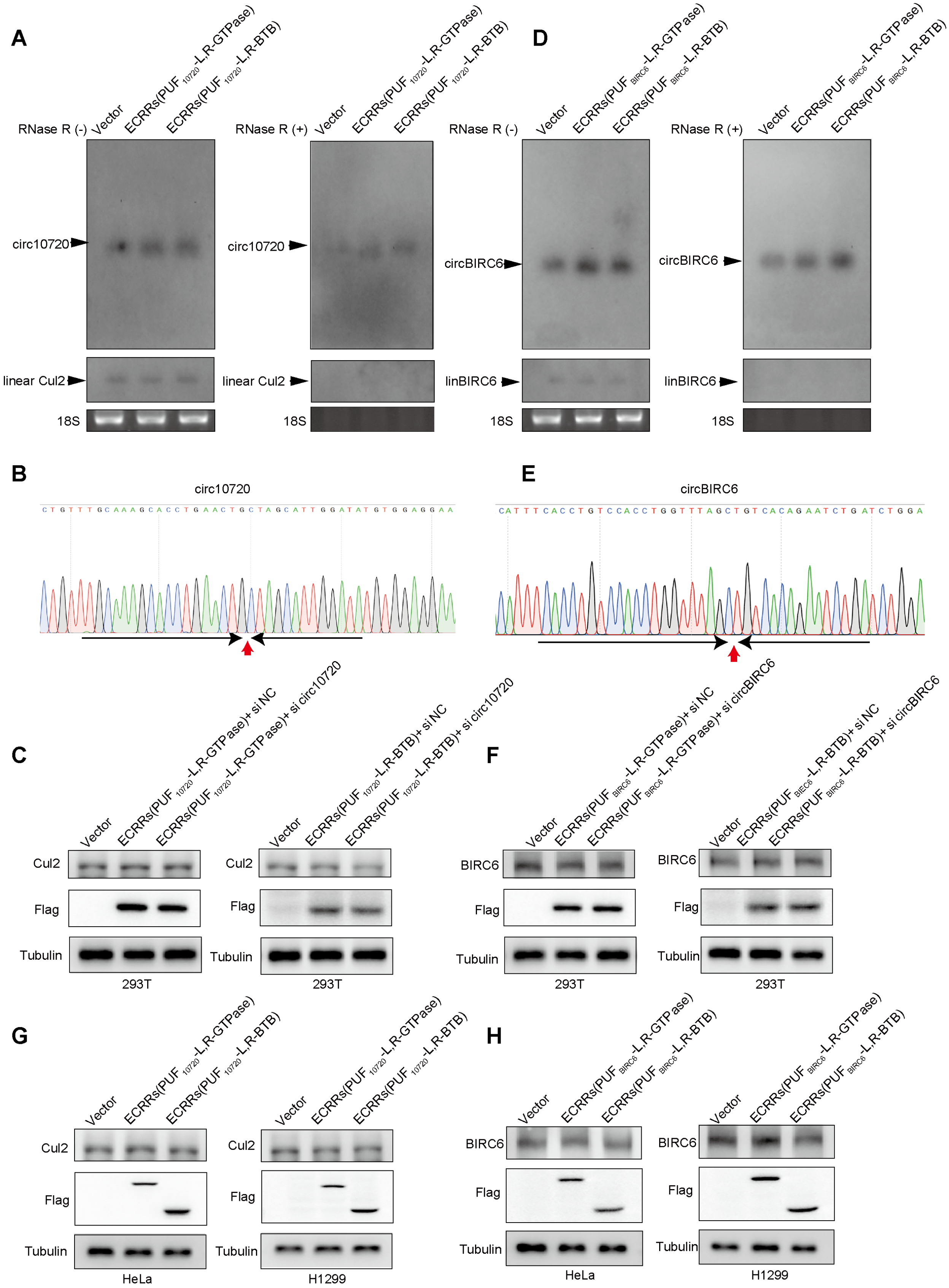

A
RNase R (-)
Vector
ECRRs(PUF 10720-L.R-GTPase)
ECRRs(PUF 10720-L.R-BTB)
RNase R (+)
circ10720
linear Cul2
18S
D
circBIRC6
linBIRC6
ECRRs(PUF BIRC6-L.R-GTPase)
ECRRs(PUF BIRC6-L.R-BTB)
B
circ10720
E
circBIRC6
C
ECRRs(PUF 10720-L.R-GTPase)+ si NC
ECRRs(PUF 10720-L.R-GTPase)+ si circ10720
ECRRs(PUF 10720-L.R-BTB)+ si NC
ECRRs(PUF 10720-L.R-BTB)+ si circ10720
Cul2
Flag
Tubulin
293T
F
ECRRs(PUF BIRC6-L.R-GTPase)+ si NC
ECRRs(PUF BIRC6-L.R-GTPase)+ si circBIRC6
ECRRs(PUF BIEC6-L.R-BTB)+ si NC
ECRRs(PUF BIRC6-L.R-BTB)+ si circBIRC6
BIRC6
G
HeLa
H1299
H

Supplementary Figure S4. ECRRs promote the production of endogenous circular RNAs. (**A**) The ECRRs(PUF$_{10720}$-L, R-GTPase) or ECRRs(PUF$_{10720}$-L, R-BTB) were used to promote the biogenesis of endogenous circ10720. The production of circ10720 and linear Cul2 RNA was examined with a northern blot assay in the absence (left) or presence (right) of RNase R. **(B)** The junction sequences of the endogenous circ10720 were verified with Sanger sequencing. (**C**) The protein levels of Cul2 and exogenously expressed ECRRs in 293T cells were determined with a western blot assay. (**D**) The ECRRs(PUF$_{BIRC6}$-L, R-GTPase) or ECRRs(PUF$_{BIRC6}$-L, R-BTB) were used to promote the biogenesis of endogenous circBIRC6. The production of circBIRC6 and linear BIRC6 RNA was examined with a northern blot assay in the absence (left) or presence (right) of RNase R. **(E)** The junction sequences of the endogenous circBIRC6 were verified with Sanger sequencing. (**F**) The protein levels of BIRC6 and exogenously expressed ECRRs in 293T cells were determined with a western blot assay. **(G)** The protein levels of Cul2, ECRR(PUF$_{10720}$-GTPase) and ECRR(PUF$_{10720}$-BTB) in HeLa and H1299 cells were examined with a western blot assay. **(H)** The protein levels of BIRC6, ECRR(PUF$_{BIRC6}$-GTPase) and ECRR(PUF$_{BIRC6}$-BTB) in HeLa and H1299 cells were examined with a western blot assay.

Supplementary Table S1

Primer	Name	Sequence	Notes
1	Gexon1f	AGTGCTTCAGCCGCTACCC	The primer pair used for testing circRNA from GFP reporter
2	Gexon3r	GTTGTACTCCAGCTTGTGCC	
3	Linear-F	ACGTAAACGGCCACAAGTTC	The primer pair used for testing linear RNA from GFP reporter
4	Linear-R	CTGAGGGCATCTTATTTGGG	
5	circScreen-F	TGTGAGTTGGATAGTTGTGGAA	The primer pair used for testing circRNA from circScreen reporter
6	circScreen-R	GGGTGTTTACCTTACGCTTC	
7	Dendra2-F	CAGAAGAAGACCCTGAAGTGG	The primer pair used for testing linear RNA from circScreen reporter
8	Dendra2-R	TTGTAGTCGCTGTCGTTGC	
9	circ-10720-F	ATGCCAACACTATTTGTGGA	The primer pair used for testing for endogenous circ-10720
10	circ-10720-R	CCCCATGGATTACTTTCTGGTTTGG	
11	Cul2-F	GGAATACGTCGAAAGAG	The primer pair used for testing cul2 mRNA
12	Cul2-R	AGCAAGGGTGCAGACTAT	
13	si circ-10720	UAUCCAAUGCUAGCAGUUCAGG	siRNA used for circ10720 knockdown
14	circBIRC6-F	TCAAGGAGACCAACTTTGGC	The primer pair used for testing endogenous circBIRC6
15	circBIRC6-R	CTGGAGTTTGCAGAGCAGTG	
16	linBIRC6-F	ATCAGGAGACCCAAGCTCAG	The primer pair used for testing for endogenous circBIRC6
17	linBIRC6-R	ACTTCCATGGCTTCCTTCTG	
19	si circBIRC6	UCAGAUUCUGUGACAGCUAAA	siRNA used for circBIRC6 knockdown
20	QKI-F	CCGACGCGTACCGGCAACGGCTCTC AGATGGTCGGGGAAATGGAAACG	The primer pair used for cloning Quaking into pCI-neo vector
21	QKI-R	CACGCGGCCGCTTAGTTGCCGGTGG CGGCTC	
22	CASTOR1-F	CCGACGCGTACCGGCAACGGCTCTC AGATGGAGCTGCACATCCTAG	The primer pair used for cloning CASTOR1 into pCI-neo vector
23	CASTOR1-R	CACGCGGCCGCTCAGGAAGCCAGGC CTTCC	
23	PKD1-F	CCGACGCGTACCGGCAACGGCTCTCA GTGCCGCGTCAACTGCTC	The primer pair used for cloning 47-178 aa of PKD1 into pCI-neo vector
25	PKD1-R	CACGCGGCCGCTTACTCACCACAGCC ACTGTC	

26	PRKAR1A-F	CCGACGCGTACCGGCAACGGCTCTCAGATGGAGTCTGGCAGTACC	The primer pair used for cloning PRKAR1A into pCI-neo vector
27	PRKAR1A-R	CACGCGGCCGCTCAGACAGACAGTGACAC	
28	DH-F	CCGACGCGTACCGGCAACGGCTCTCAGAAGCGACAAGGATACATCC	The primer pair used for cloning DH domain of ITSN1 into pCI-neo vector
29	DH-R	CACGCGGCCGCTTACCCTTCGTTCACCTGG	
30	ZBTB18-F	CCGACGCGTACCGGCAACGGCTCTCAGATGTGTCCTAAAGGTTATGAAG	The primer pair used for cloning ZBTB18 into pCI-neo vector
31	ZBTB18-R	CACGCGGCCGCTTATTTCCAAAGTTCTTG	
32	BTB-F	CCGACGCGTACCGGCAACGGCTCTCAGTGTGACTGCACTGTTCTG	The primer pair used for cloning the BTB domain of ZBTB18 into pCI-neo vector
33	BTB-R	CACGCGGCCGCTTAGTCTTTGAACTGGAGTTTC	
34	HNRNPA1-F	CCGACGCGTACCGGCAACGGCTCTCAGATGTCTAAGTCAGAGTCTCC	The primer pair used for cloning HNRNPA1 into pCI-neo vector
35	HNRNPA1-R	CACGCGGCCGCTTAAAATCTTCTGCCACTGCC	
36	UP1-R	CACGCGGCCGCTTACTCTTGCTTTGACAGGGCTTTTC	The primer with HNRNPA1-F used for cloning the UP1 domain of HNRNPA1
37	GTPase-F	CCGACGCGTACCGGCAACGGCTCTCAGACATATGAATGGAGCTCAGAAG	The primer pair used for cloning the GTPase domain of ATL1 into pCI-neo vector
38	GTPase-R	CACGCGGCCGCTTAGGTACGAGCTGCATGGAAG	
39	flagnls-F	CCGCTCGAGCCATGGACTACAAGGACGACGATGACAAGCCCAAGAAAAAGAGGAAG	The primer pair used for cloning FLAG-NLS-PUF into pCI-neo vector
40	EcoPUF-F	CCCAAGAAAAAGAGGAAGGTAGAATTCGGCCGCAGCCGCCTTTTGGAAG	
41	PUF-Mlu-R	CCGACGCGTCCCTAAGTCAACACCGTTCTT	
42	pLVX -F	CCGCCCGGGCCATGGACTACAAGGACGACG	The primer pair with GTPase-R or BTB-R used for clone $ECRR_{10720/\ BIRC6}$ into pLVX-Puro vector.
43	HAnls-F	ccgCTCGAGccatgTACCCATACGACGTCCCAGACTACGCTCCCAAGAAAAAGAGGAAG	The primer with reverse primers of different dimerized protein or domains used for cloning HA-tag ECRRs into pCI-neo vector.

44	T7-circGFP-R	TAATACGACTCACTATAGGGTTACTTGTAC AGCTCGTCC	Northern probes
45	GFP-probe-F	ATGGTGAGCAAGGGCGAGG	Northern probes
46	T7-linear-R	TAATACGACTCACTATAGGG GCTGTCCCAGTCCTCTATCC	Northern probes
47	linear-probe-F	ATCACATCTTTAAGCCTTCCAT	Northern probes
48	T7-circBIRC6-R	TAATACGACTCACTATAGGGCT GGAGTTTGCAGAGCAGTG	Northern probes
49	BIRC6-probe-F	TCAAGGAGACCAACTTTGGC	Northern probes
50	T7-preBIRC6-R	TAATACGACTCACTATAGGGAC CAAGCCTGGCCTAATTCT	Northern probes
51	T7-cul2-R	TAATACGACTCACTATAGGGGTGGACACA GCACGGAGTAAG	Northern probes
52	cul2-probe-F	CGTGGTGGAGAAGACCCAAACC	Northern probes
53	T7-circ10720-R	TAATACGACTCACTATAGGGTGTTTCAGT CAGAAAGGGAGA	Northern probes
54	circ10720-probe-F	GCCAACACTATTTGTGGAGTC	Northern probes
